# The Relationship between Structure and Performance of Different Polyimides Based on Molecular Simulations

**DOI:** 10.3390/polym15030646

**Published:** 2023-01-27

**Authors:** Peng Zhang, Yadong Dai, Hansong Liu, Botao Dong, Yilun Yao, Jinsong Sun, Tao Yang, Xiangyu Zhong, Jianwen Bao

**Affiliations:** 1 Science and Technology on Advanced Composites Laboratory, AVIC Composite Technology Center, AVIC Manufacturing Technology Institute, Beijing 101300, China; 2 Neotrident Co., Ltd., Beijing 100191, China

**Keywords:** polyimide, molecular simulation, cross-link, viscosity, glass-transition temperature

## Abstract

A polyimide (PI) molecular model was successfully constructed to compare the performance of PIs with different structures. In detail, the structure of the cross-linked PI resin, the prepolymer melt viscosity, and the glass-transition temperature (T_g_) were investigated using molecular simulations. The results indicate that benzene ring and polyene-type cross-linked structures dominate the properties of the PIs. Moreover, the prepolymer melt viscosity simulations show that the 6FDA-APB and the ODPA-APB systems have a low viscosity. The results for the T_g_ and the distribution dihedral angle reveal that the key factor affecting bond flexibility may be the formation of a new dihedral angle after cross-linking, which affects the T_g_. The above results provide an important reference for the design of PIs and have important value from the perspective of improving the efficiency of new product development.

## 1. Introduction

Polyimides (PIs), which perform excellently and are widely used, are attracting increasing attention from researchers [1,2,3,4]. Their performance-related advantages include high mechanical strength, high elastic modulus, good chemical resistance, and high thermal resistance [5,6,7,8,9,10]. Thus, PIs are widely used in many fields, including aeronautical, electronics, and chemical industries [11,12,13,14,15]. Among these, thermosetting PIs are widely utilized in the field of aerospace as a structural matrix [16,17,18,19,20]. However, there are many types of thermosetting PIs, and their different structures determine different performance characteristics. In terms of traditional research methods, researchers try to synthesize various types of materials and then compare their properties. However, this method is inefficient. Moreover, the conditions of synthesis must be precisely controlled; otherwise, errors will be introduced into the process of comparing performance.

With the development of computer technology and simulation software, simulation technology has been shown to greatly reduce the cost of material development and improve work efficiency [21,22,23,24,25,26]. A molecular dynamic simulation optimization experiment showed that an efficiency ratio of running time that tended to balance judgment achieved the highest value of 36.96% [27].

Specifically, for thermosetting PI resin systems, Mo et al. [28] built an initial periodic unit cell of PI and calculated its stiffness matrix and mechanical parameters. Qiu et al. [29] focused on thermoplastic PIs with a low dielectric constant based on 4,4’-(4,4’-Isopropylidenediphenoxy)diphthalic anhydride (BPADA)-2,2-Bis(4-(4-aminephenoxy)phenyl)propane (BAPP). The results from the molecular simulations indicated that anhydride monomers, which contained lateral methyl groups, transformed PIs to become thermoplastic. Lin et al. [30] investigated the effect of nano-zinc oxide (ZnO) on the surface binding energy of PI/ZnO composites and obtained a relationship between the surface binding energy of the PI/ZnO composites and the nano-ZnO radius, as well as the maximum of the surface binding energy. Wen et al. [31] synthesized a novel PI using 4-Amino-N’-(4-aminobenzoyl)benzohydrazide (AAPDA) and pyromellitic dianhydride (PMDA). Molecular simulations were used to determine the number of cavities in the PI films and to analyze their size distributions. However, most studies have focused on a specific molecular structure. A transverse comparison of PI properties with different chemical structures and related research on material properties of interest in engineering applications (viscosity and heat resistance) still need to be investigated.

In this study, the molecular models of two series of phenylethynyl end-capped thermosetting PI resins were constructed by polymerization with different types of dianhydride and diamine through a molecular-structure design. The structure of the cross-linked polyimide resin, the prepolymer melt viscosity, and the glass-transition temperature (T_g_) were calculated in a simulation software. The comparison of these results can provide a reference for the molecular-structure design of PI resin. This simulation method is expected to be popularized in engineering applications to improve the development efficiency of new material systems.

## 2. Molecular Models of Polyimide Resin System and Simulation Details

The simulation process is divided into three parts: First, an amorphous molecular model of the PI resins was constructed. Then, according to the cross-linking reaction mechanism, a 3D polymer network structure was formed dynamically using a Perl script in the Materials Studio software. Finally, the shear viscosity was predicted based on the amorphous model and the T_g_ was predicted based on the cross-linking model. The molecular simulation software used was Materials Studio 2017 developed by BIOVIA which is located in Paris, France. In our work, the COMPASSII force field (condensed-phase optimized molecular potential for atomistic simulation studies) was used to describe the interaction between atoms.

### 2.1. The Amorphous Molecular Model

**Figure 1:**
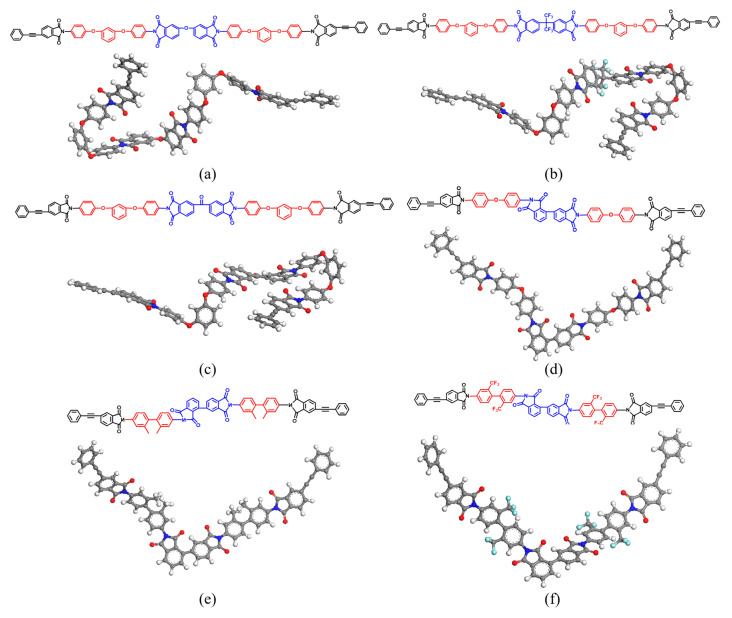
(**a**–**f**) Molecular structures of the oligomers.

In order to design higher-performance PI resins, two series of phenylethynyl end-capped thermosetting PI resins were constructed by polymerization with different types of dianhydride and diamine through a molecular-structure design. The three systems of series 1 comprise diamine 1,3-Bis(4-aminophenoxy)benzene (1,3,4’-APB) and three different types of dianhydride (4,4-Oxydiphthalic anhydride (ODPA), 2,2-Bis(3,4-anhydrodicarboxyphenyl)-hexafluoro propane (6FDA), and 3,3’,4,4’-Benzophenone tetracarboxylic dianhydride (BDTA)). The three systems of series 2 comprise dianhydride 2,3,3’,4’-Biphenyltetracarboxylic dianhydride (α-BPDA) and three different diamines (4,4’-Oxydianiline (4,4’-ODA), 2,2’-Bis(trifluoromethyl) benzidine (TFMBZ), and 2,2’-dimethyl-4,4’-diaminobiphenyl (DMBZ)). When considering the rationality of the PI structures and the calculation resources, the polymerization degree of the PIs is one, and the structures are shown in Figure 1.

**Figure 2:**
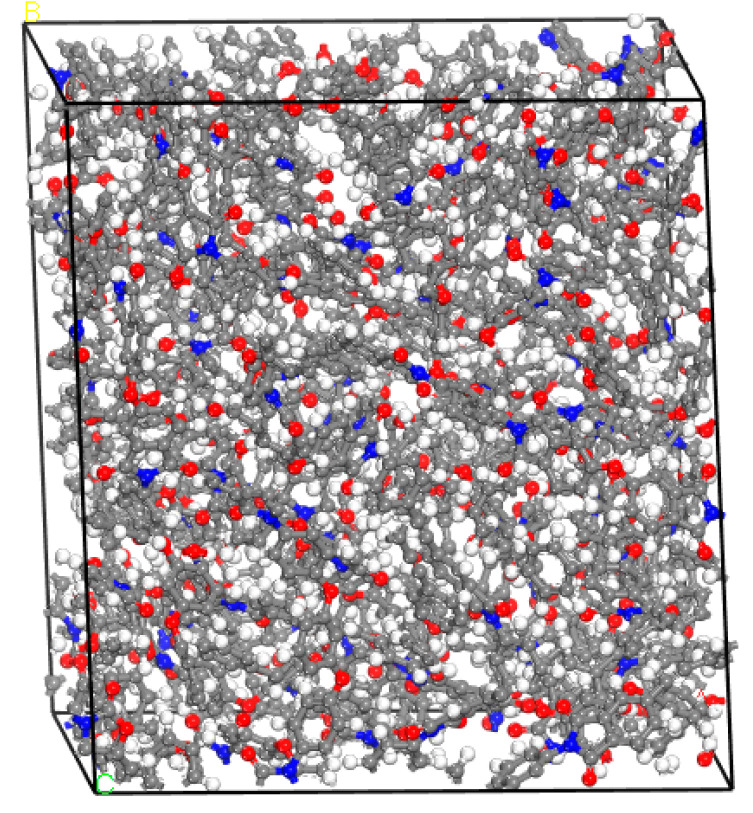
The amorphous structure of the ODPA-APB PI resin.

Using the Amorphous Cell module based on the Monte Carlo method, a three-dimensional disordered stacking structure model was built on the basis of a single molecular structure. In this study, six amorphous structures were built based on the six molecular structures in Figure 1. Each structure contains 30 molecular chains, and the initial system density was set to 0.5 g/cm^3^. In order to obtain a structure that can represent the real system, the amorphous structure was optimized using the Forcite plus module based on the geometry optimization criteria (energy is 1.0 × 10^−4^ kcal/mol, force is 0.005 kcal/mol/Å, and displacement is 5.0 × 10^−5^Å). Because this optimized structure might, however, still be in a local energy minimum state, this was relaxed through the NVT molecular dynamics for 250 ps and the NPT molecular dynamics for 250 ps. Finally, a full optimization for cell parameters and atomic coordinates was performed by using molecular mechanics and molecular dynamics, and the equilibrium density of each structure was obtained. Figure 2 shows the amorphous structure of the constructed ODPA-APB PI resin; the structure of other systems is similar.

### 2.2. Construction of Cross-Linked Polyimide Resin

**Figure 3:**
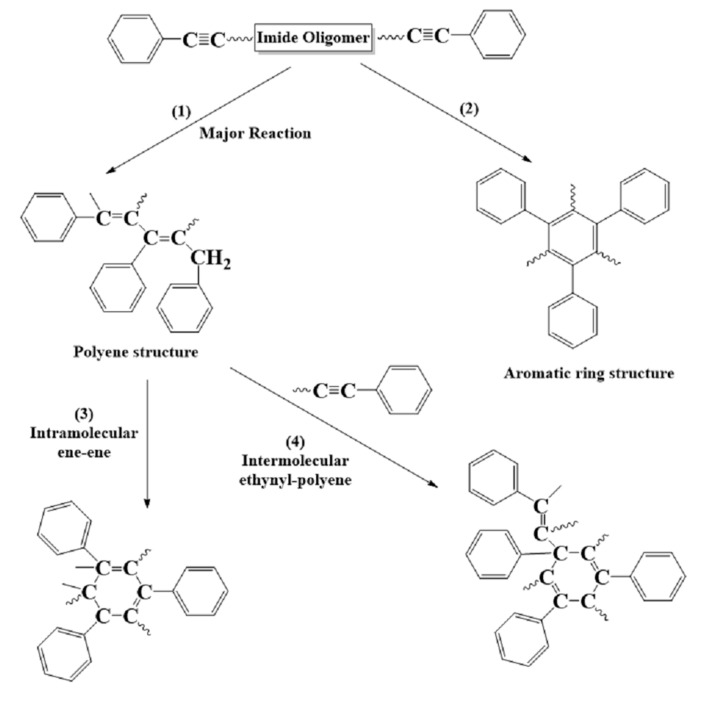
Proposed phenylethynyl curing process.

The equilibrious amorphous structure is cross-linked according to the curing reaction mechanism to form a high degree of cross-linking 3D polymer network structure. It is well understood that four principal reactions take place in a phenylethynyl end-capped thermosetting PI resin system [32], as illustrated in Figure 3. The major cure reaction is the ethynyl-to-ethynyl reaction to form double bonds or polyene structures (chain extension). Based on the above reaction mechanism, the cross-linked PI resin was realized by a self-made cross-linking program. The specific process is as follows.

Step 1: First, the cut-off radius and the preset cross-linking degree are determined. The cut-off radius is used as the criterion for deciding whether the reacting atoms can be cross-linked, and the initial cut-off distance is set as 4 Å.

Step 2: Then, the distance of the C atom in all alkynyl groups in the amorphous system is calculated and compared with the cut-off radius. If it is less than the cut-off radius, the cross-linking reaction is completed, according to the reaction mechanism, to generate a new cross-linking bond; otherwise, no cross-linking bond is generated. The whole process follows the principle of one cross-linking of the same atom at a time and the priority of unsaturated atoms to judge the reactive cross-linking sites. After adjusting the chemical bond, we recalculate the type of force field and the charge of atoms in the system.

In order to generate new cross-linking bonds for the new model, the structural relaxation, 100 ps NVT dynamic calculation, and 100 ps NPT dynamic calculation were carried out. 

Step 3: Each distance was run 15 cycle times until the set upper limit of cycle times or the preset cross-linking degree is reached. 

Step 4: The cut-off distance is increased by 1 Å and step 2 is repeated until the cut-off radius distance is greater than 6 Å or reaches the preset cross-linking degree, then the crosslinking process is completed. In order to give priority to the cross-linking of unsaturated atoms formed in the cross-linking process, the cross-linking distance of unsaturated atoms will be increased by 2 Å over the actual set distance.

### 2.3. Viscosity Calculation

Melt viscosity has an important influence on the processing properties of a resin system. In order to better evaluate the processing properties of the PI resin, based on the Green–Kubo linear response theory [33,34,35], the shear viscosity is calculated using equilibrium molecular dynamic simulations. The viscosity is calculated by integrating the stress autocorrelation function, as illustrated in Equation (1).

(1) $$\eta =\frac{V}{\mathrm{kT}}\overset{\infty }{\underset{0}{\int }}{<P}_{\alpha \beta }\left(t\right)P_{\alpha \beta }\left(0\right)>\mathrm{dt}\;$$

where *V* is the volume of the system; *k* is the Boltzmann constant; *T* is the temperature; *P_αβ_* refers to the three equivalent off-diagonal components of the instantaneous pressure tensor; and < > represents the statistical average.

In order to obtain shear viscosity data that are comparable to the experiment, it is necessary to fully relax the melt structure to ensure the elimination of the stress correlation of the model. Therefore, a series of molecular dynamic calculations were carried out to obtain the shear viscosity. In order to obtain the global optimal structure, a simulated annealing molecular dynamic calculation was carried out. Then, the lowest energy structure of annealing was extracted, and the molecular dynamic calculation of the NPT ensemble was carried out to obtain a reasonable density of the system. Finally, the molecular dynamic simulation of the NVT ensemble was carried out up to the dozens or even hundreds of nanoseconds to calculate the shear viscosity. The temperature and the pressure were controlled through a Nosé Hoover Langevin thermostat and a Berendsen barostat [36], respectively. The long-range electrostatic interactions and the van der Waals interactions [37] were calculated using the group-based method. For the electrostatic and van der Waals interactions, a cutoff radius of 18.5 Å was set. The charge group was divided according to the divide-and-conquer method [38,39], and the allowable charge deviation was less than 0.01 e.

Based on the obtained stable dynamic trajectory, the stress autocorrelation function was analyzed by using the Forcite Plus analytical tool, and the appropriate origin step and the length values were selected for each system. A set of shear viscosity data can be obtained with different length settings, and the curve of shear viscosity with length can be obtained. Because the trajectory file to be analyzed was relatively large, this part of the work was mainly completed through home-written Perl scripts. According to the results of Nevins et al. [40] and the calculated results of this paper, the shear viscosity would increase with an increase in the length of the chains, and the shear viscosity value would appear at an approximately stable level, which would correspond to the shear viscosity of the system. Therefore, the shear viscosity of different PI resin systems was obtained by this method. In order to obtain stable and accurate simulated results, the final shear viscosity data are the average of five melt structure models with the same composition.

### 2.4. Glass-Transition Temperature Calculation

The vitrification transition of amorphous polymers refers to the transition process between the glassy and rubber states of polymer materials, and the transition temperature is called T_g_. Glassy amorphous polymers exhibit hard and brittle mechanical states at temperatures lower than T_g_. Polymer materials in a glass state have stable mechanical properties in the process of use, and T_g_ is usually used as the upper-temperature limit of this kind of material. During the vitrification transformation, the thermal, mechanical, electrical, and other properties of polymer materials will change, so T_g_ is of great significance for the selection and application of polymer materials.

Based on the cross-linking models of the PI systems, the density at each temperature was simulated. Then, a density temperature curve was obtained, and the inflection point of the curve, that is, the transition point of density with temperature change rate, was obtained by linear fitting so as to obtain the T_g_ of the model. In order to obtain a more accurate density value, it is necessary to fully relax the cross-linking model. Therefore, a 300 ps dynamic simulation under the NVT ensemble was carried out at each temperature point, followed by a 300 ps dynamic simulation under the NPT ensemble. In the simulation, the temperature points of the ODPA-APB, the BTDA-APB, and the 6FDA-APB systems were 1000~300 K. The temperature range used to describe the variation in density and dihedral angle with temperature was 800~300 K. The temperature points of the α-BPDA-4,4-ODA, the α-BPDA-DMBZ, and the α-BPDA-TFMBZ systems were 1000~475 K. The temperature range used to describe the variation in density and dihedral angle with temperature was 900~475 K. The temperature and the pressure were controlled through a Nosé Hoover Langevin thermostat and a Berendsen barostat, respectively. The density and dihedral angle distribution data of the system were obtained based on an analysis of the trajectory file generated by the NPT ensemble. During the calculation, one frame’s output was every 1.5 ps, making a total of 200 frames. In order to obtain stable and accurate simulated results, the final density and dihedral angle distribution of the system is the average of the parallel-simulation results of five melt structure models with the same composition.

## 3. Results and Discussion

### 3.1. Structure of Cross-Linked Polyimide Resin

**Figure 4:**
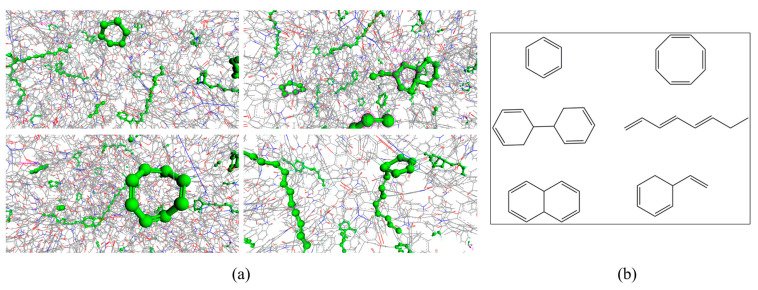
(**a**) The partial structures of the ODPA-APB cross-linked PI resin: in order to distinguish the changes of the molecular model before and after cross-linking, the linear model is used to represent the atoms that do not participate in the crosslinking reaction, and the spherical model is used to represent the cross-linked covalent bond formed. (**b**) Important structural fragments formed by the cross-linking of three bond carbon atoms in the phenylethynyl group.

In this study, the cross-linked structure of the ODPA-APB obtained is taken as an example, as shown in Figure 4. The figure shows the partial structures and the important structural fragments formed by the cross-linking of three-bond carbon atoms in the phenylethynyl group. These structural fragments are randomly distributed in different models, and the number of structural fragments of the benzene ring and polyene type is the largest. This result is basically consistent with the expected result. In the 30 models studied, the cross-linking degree (the ratio of the number of cross-linking atoms to the number of cross-linking sites) is distributed between 85% and 95%.

### 3.2. Prepolymer Melt Viscosity

The data of the shear viscosity of the ODPA-APB, the 6FDA-APB, the BTDA-APB, the α-BPDA-4,the 4-ODA, the α-BPDA-DMBZ, and the α-BPDA-TFMBZ are shown in Table 1.

The order of the shear viscosity obtained by the theoretical simulation at 573.15 K from large to small is as follows: α-BPDA-4,4-ODA > α-BPDA-TFMBZ > BTDA-APB > α-BPDA-DMBZ > 6FDA-APB > ODPA-APB. The order of the shear viscosity measured experimentally is as follows: α-BPDA-4,4-ODA > α-BPDA-TFMBZ > ODPA-APB > 6FDA-APB. The theoretical calculation data of the 6FDA-APB and the α-BPDA-TFMBZ systems are close to the experimental data, and the viscosity value trend of the system is the same, which shows that the theoretical method can predict the prepolymer melt viscosity of the materials and is helpful for a rapid screening of the materials.

### 3.3. Glass-Transition Temperature

**Figure 5:**
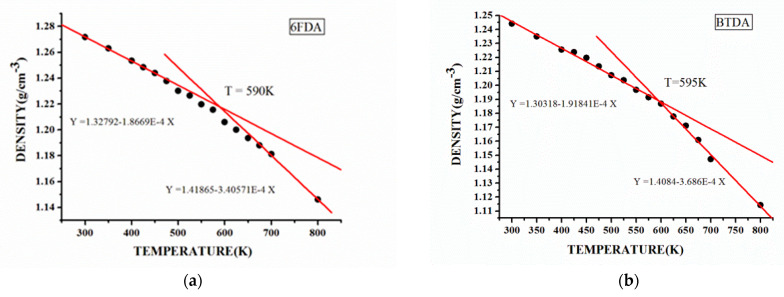
The linear fitting results of density variation with temperature of each simulated model. (**a**) 6FDA-APB, and (**b**) BTDA-APB.

By analyzing the density of each model at different temperatures, the *ρ*-*T* curves shown in Figure 5 and Figure S1 (Supplementary Materials) were obtained, and T_g_ values were obtained according to the linear fitting of the curve. It can be seen from the data in Table 2 that these simulated T_g_ values are in good agreement with the experimental results. Among them, the T_g_ values of the 6FDA-APB, the BTDA-APB, and the ODPA-APB are very close, but the T_g_ values of the α-BPDA -4,4-ODA, the α-BPDA-DMBZ, and the α-BPDA-TFMBZ are significantly different. This shows that the effect of the three different anhydride structures on the PIs’ T_g_ is not obvious, but the three different diamine structures have an important effect on the PIs’ T_g_.

#### 3.3.1. Δ*ρ*-*T* Curve Analysis

**Figure 6:**
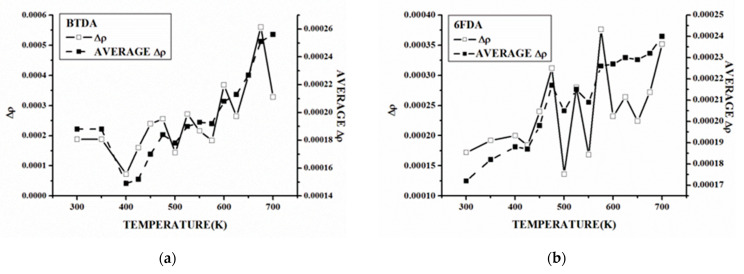
The Δ*ρ*-*T* curves and Δ*ρ* mean value curves with temperature of each system: (**a**) BTDA-APB and (**b**) 6FDA-APB.

The *ρ*-*T* curves in Figure 5 and Figure S1 show that the α-BPDA-TFMBZ and the α-BPDA-DMBZ have obvious points where the density changes with temperatures of 800 K and 825 K, which are significantly different from the α-BPDA-4,4-ODA. In order to better understand the relationship between the molecular structure and the T_g_ and to more intuitively reflect the characteristics of the Δ*ρ*-*T* curve, it is assumed that the system density between the two temperature points in the simulation matches the linear change, and the change rate in the density Δ*ρ* is defined as illustrated in Equaiton (2):

(2) $${\Delta \rho }_{i}=\frac{\rho_{i}-\rho_{i+1}}{T_{i+1}{-T}_{i}}\;$$

when *T_i_* < *T*_*i*+1_, the curve is obtained by analyzing the relationship between the density change rate and the temperature, as shown in Figure 6 and Figure S2.

The analysis of the Δ*ρ*-*T* curves of each system shows that Δ*ρ* has a relatively stable temperature range at a low-temperature stage, which indicates that the density basically meets the law of linear change with the temperature at this stage. As the temperature increases, Δ*ρ* will oscillate, but the Δ*ρ* average value statistics show that the Δ*ρ* oscillation in the low-temperature stage often cannot effectively increase the Δ*ρ* value. The Δ*ρ* average value statistics of the BTDA-APB in Figure 6 show that, even at 575 K, the Δ*ρ* average value increment, when compared to the initial value, is only 6 % of the maximum temperature of 700 K.

In the BTDA-APB, the ODPA-APB, and the α-BPDA -4,4-ODA, Δ*ρ* oscillates obviously near the upper limit of the simulated temperature, and the average value of Δ*ρ* increases obviously, but there is no obvious change in Δ*ρ* at the corresponding temperature of the *ρ*-*T* curve. Because the Δ*ρ*-*T* curve at each temperature point of Δ*ρ* only reflects the *ρ* in the next temperature range of speed, Δ*ρ* appears to substantially increase but also greatly reduce, as shown in the BTDA-APB, the ODPA-APB, and the α-BPDA -4,4-ODA, although this is not necessarily able to cause Δ*ρ* to significantly increase, with a statistically larger temperature range for the Δ*ρ* value. We found that the Δ*ρ* value of the BTDA-APB in the range of 625~800 K is 3.62 × 10^−4^g/cm^−3^ K, which is very close to the Δ*ρ* value of 3.68 × 10^−4^g/cm^−3^ K at 600 K, indicating that the Δ*ρ* value increases compared with that at 600 K. The Δ*ρ* oscillation of the BTDA-APB in the range of 625~800 K has little effect on the Δ*ρ* increase, which is in accordance with the characteristics of the *ρ*-*T* curve. The Δ*ρ* oscillations in the range of 650~800 K for the ODPA-APB and 725~900 K for the α-BPDA-4,4-ODA also have the same characteristics. Therefore, the temperature at which the average Δ*ρ* value increases significantly will have a significant impact on T_g_.

#### 3.3.2. Statistical Analysis of Dihedral Angle Variation with Temperature

##### Δ*P*-*T* Curve Acquisition and Analysis

In order to obtain the relationship between dihedral angle variation and temperature, the absolute value of the difference in the dihedral angle distribution probability at different temperatures was obtained by using dynamic simulation.

**Figure 7:**
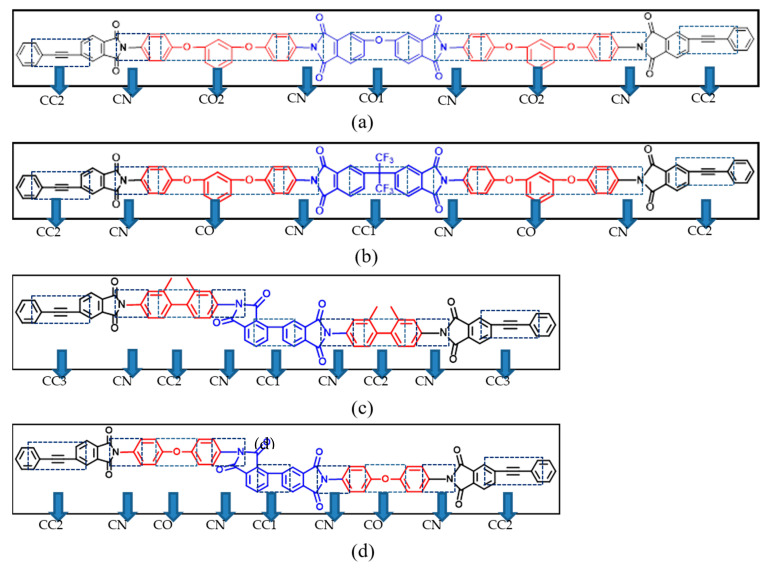
Division of dihedral angle: (**a**) ODPA-APB; (**b**) 6FDA-APB; (**c**) α-BPDA-DMBZ; and (**d**) α-BPDA-4,4-ODA.

The dihedral angles of the different systems are divided according to the different positions of atoms that constituted the dihedral angles in the system, as shown in Figure 7; the representative ODPA-APB, 6FDA-APB, α-BPDA-DMBZ, and α-BPDA-4,4-ODA are selected here for illustration. The partition in the BTDA-APB is the same as the 6FDA-APB, and the partition in the α-BPDA-TFMBZ is the same as the α-BPDA -DMBZ.

These dihedral angles can be roughly divided into two categories, one of which is the dihedral angle CC3 or CC2 at the phenylacetylene group position, representing various dihedral angles at the cross-linking site, including the newly generated dihedral angles in the cross-linking process. The other is the intramolecular dihedral angles at two cross-linking sites, which mainly include various dihedral angles in the diamines and anhydrides.

On this basis, the dihedral angle distribution with temperature can be obtained by selecting the angle distribution probability at the lowest temperature as the only reference. The definition of *ΔP* is satisfied, as illustrated in Equation (3):

(3) $$\Delta P=\frac{\sum_{i=1}^{N}\left|P_{i,T}{-P}_{{i,T}^{\prime }}\right|}{N}$$

when N = $\frac{360{}^{\circ}}{B}$ and *B* = 1°, *P_i,T_* represents the probability of distribution of the dihedral angle on the *i* angle value at temperature *T*, and *B* represents the smallest group distance of the statistical dihedral angle distribution, namely the angle interval. Δ*P* is the change in dihedral angle distribution relative to *T’* at temperature *T*. When *T’* is fixed as the minimum value of the simulated temperature (300 K or 475 K), Δ*P* and the corresponding Δ*P*-*T* curves at other temperatures are obtained to describe the change in the dihedral angle distribution with temperature.

**Figure 8:**
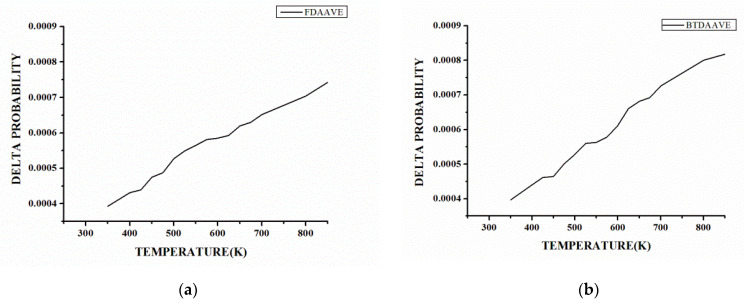
Temperature variation in the dihedral angle distribution in the main chain structure: (**a**) 6FDA-APB, and (**b**) BTDA-APB.

It can be seen from the Δ*P*-*T* curve in Figure 8 and Figure S3 that Δ*P* generally increases with an increase in temperature because the dihedral angle is activated gradually with the increase in temperature, and the angle distribution range keeps changing. However, Δ*P* does not increase monotonically and linearly. In some temperature ranges, Δ*P* remains relatively stable. As shown in Figure 8a, the dihedral angle of the 6FDA-APB remains relatively stable in the temperature ranges of 400~425 K, 450~475 K, 575–625 K, and 650~675 K. The results indicate that Δ*P* will reach a certain extreme value in a certain temperature range, and only after reaching or exceeding a certain temperature, the distribution of the dihedral angle will change further. These "platforms" are the embodiment of the fact that the dihedral angle torsion needs to overcome a certain energy barrier. On the other hand, the distribution of such temperature ranges is not consistent in different systems, and even for the same system, the variation in Δ*P* with *T* is different in different temperature ranges. As shown in Figure 8b, the variation rate of Δ*P* with *T* in the range of 575~625 K of the BTDA-APB is significantly higher than that in other temperature ranges. Therefore, it can be found that the Δ*P*-*T* curve can reflect not only the basic characteristics of the dihedral angle distribution changing with temperature but also the difference in the dihedral angle change among different systems.

##### Analysis of the Relationship between T_g_ and Change in Dihedral Angle

The relationship between density increments and temperature shows that the temperature of an increased Δ*ρ* is consistent with that of an increased Δ*v*. When Δ*ρ* increases, the value of Δ*v* of the corresponding dihedral angle increases, which directly reflects the influence of the change in the dihedral angle on the density of the system. A faster change in the dihedral angle with temperature also leads to an increase in the rate of change in the density with temperature.

T_g_ is a macroscopic expression of the gradual activation of the dihedral angle with increasing temperature, while Δ*P* and Δ*v* are both microscopic descriptions of the activation of the dihedral angle. *ΔP* represents the angular distribution of a dihedral angle at different temperatures, and Δ*v* represents the rate at which the angular distribution of a dihedral angle changes with temperature. The larger the Δ*v* is, the more significant the activation of the dihedral angle is. A smaller Δ*v* indicates that the activation of the dihedral angle is limited and changes slowly with the increase in temperature. The extreme case of when Δ*v* ≈ 0 indicates that the dihedral angle does not change significantly with temperature, corresponding to the "platform" in Δ*P*-*T*. Therefore, the temperature corresponding to the increase in Δ*v* is more closely correlated with the T_g_. At the same time, the Δ*P* between the two temperature points in the simulation changes linearly, and the change rate Δ*v* is defined as illustrated in Equation (4):

(4) $${\Delta v}_{i}=\frac{{\Delta P}_{i+1}{-\Delta P}_{i}}{T_{i+1}{-T}_{i}}\;$$

**Figure 9:**
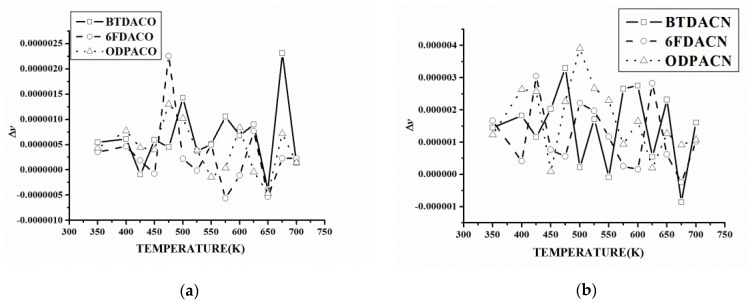
Comparison of *Δv* values of CN and CO dihedral angles in the BTDA-APB, the 6FDA-APB, and the ODPA-APB: (**a**) CO, and (**b**) CN.

The Δ*v* values of CN and CO in the adjacent diamine structures in the BTDA-APB, the 6FDA-APB, and the ODPA-APB systems were analyzed, and the results are shown in Figure 9. Generally, the structures of the BTDA-APB, the 6FDA-APB, and the ODPA-APB show that the rigidity of the anhydride chain segment in the BTDA-APB is the strongest because the carbonyl group forms a conjugate structure with the adjacent benzene ring, while the the rigidity of ODPA-APB is the weakest due to the addition of oxygen atoms to the ether bond increases the flexibility of the segment. These results show that the CN and CO of the BTDA-APB still maintain large Δ*v* values in the temperature range of 600 K and above. The CN of the 6FDA-APB is similar to that of the BTDA-APB, but the Δ*v* value of the CO decreases obviously. Compared with the BTDA-APB, the Δ*v* values of the CO and CN in the ODPA-APB decrease significantly. As mentioned above, the larger the Δ*v* is, the more significant the activation of the dihedral angle is, and the more rapid the change is with the increase in temperature. A smaller Δ*v* indicates that the activation of the dihedral angle is limited and changes slowly with the increase in temperature. The BTDA-APB, the 6FDA-APB, and the ODPA-APB have the same diamine structure, and the torsion of the CN and CO dihedral angle is mainly affected by the structure of the adjacent anhydride. The Δ*v* values in the figure indicate that the torsion of the CN and CO in the BTDA-APB at a low temperature is obviously limited, which is related to the rigidity of the structure of the BTDA-APB anhydride. In sharp contrast, the CN and CO change slowly with temperature in the high temperature section due to the obvious weakening of the ODPA-APB’s restriction on them. The rigidity of the 6FDA-APB is in the middle. Because CN molecules are closer to each other, the restriction on the CN is similar to that of the BTDA-APB, while for the DISTANT CO, it is basically the same as the ODPA-APB. This also confirms the previous statement that the Δρ increase at 700 K in the 6FDA-APB is related to the dihedral angle CC1(6FDA-APB) in the anhydride.

To summarize, the T_g_ and molecular structure of a PI have a close relationship, the softness of the dihedral angle influences the movement of the polymer chain, and the characteristics of the groups for the dihedral angle reversed by biphenyl have an obvious limiting effect. At the same time, the group size and the charge from the perspective of the space steric hindrance and coulomb make it harder to reverse the dihedral angle. Therefore, the formation of a new dihedral angle after cross-linking may be an important factor affecting bond flexibility, thus affecting the T_g_ of the whole system.

## 4. Conclusions

In this work, an amorphous molecular model of PIs was established, containing four kinds of dianhydride and four kinds of diamine. According to the cross-linking reaction mechanism, a 3D polymer network structure was formed dynamically. The cross-linking structure of three-bond carbon atoms in the phenylethynyl group showed that the number of structural fragments of the benzene ring and polyene type was the largest. The shear viscosity was predicted based on the amorphous model, and the theoretical calculation data showed that the viscosity value trend of the system was predictable. The T_g_ was predicted based on the cross-linking model, and the simulated T_g_ values were in good agreement with the experimental results. The analysis of the relationship between the T_g_ and the dihedral angle change revealed that the formation of a new dihedral angle after crosslinking might be an important factor affecting bond flexibility, thus affecting the T_g_ of the whole system.

## Figures and Tables

**Table 1:** The system density of the prepolymerized PI resins was calculated theoretically, and the prepolymer melt viscosity of the PI resins was measured experimentally and calculated theoretically at 573.15K.

System	Density _Theo_ (g/cm^3^)	Viscosity _Theo_ (Pa·s)	Viscosity _Exp_ (Pa·s)
ODPA-APB	1.169	0.179	0.8360
6FDA-APB	1.213	0.331	0.4968
BTDA-APB	1.202	1.707	~
α-BPDA-4,4-ODA	1.179	7.258	3.088
α-BPDA-DMBZ	1.176	1.385	~
α-BPDA-TFMBZ	1.276	2.345	2.676

**Table 2:** T_g_ of each model.

System	6FDA-APB	BTDA-APB	ODPA-APB	α-BPDA -4,4-ODA	α-BPDA -DMBZ	α-BPDA-TFMBZ
T_g_ /_Theo_(K)	590	595	580	686	771	723
T_g_ /_Exp_(K)	593	618	569	660	773	720

## Data Availability

The data presented in this study are available from the corresponding author upon request. The data are not publicly available due to the fact that they are also part of an ongoing study.

## Supplementary Materials

The following supporting information can be downloaded at https://www.mdpi.com/article/10.3390/polym15030646/s1, Figure S1: Linear fitting results of the density variation with temperature of each simulated model; Figure S2: Δ*ρ*-*T* curves and Δ*ρ* mean value curves with temperature of each system; Figure S3: Temperature variation in the dihedral angle distribution in the main chain structure.

## References

[1] Liaw D.J., Wang K.L., Huang Y.C., Lee K.R., Lai J.Y., Ha C.S. (2012). Advanced polyimide materials: Syntheses, physical properties and applications. Prog. Polym. Sci..

[2] Zhuang Y.B., Seong J.G., Lee Y.M. (2019). Polyimides containing aliphatic/alicyclic segments in the main chains. Prog. Polym. Sci..

[3] Sanaeepur H., Amooghin A.E., Bandehali S., Moghadassi A., Matsuura T., Van der Bruggen B. (2019). Polyimides in membrane gas separation: Monomer’s molecular design and structural engineering. Prog. Polym. Sci..

[4] Ni H.J., Liu J.G., Wang Z.H., Yang S.Y. (2015). A review on colorless and optically transparent polyimide films: Chemistry, process and engineering applications. J. Ind. Eng. Chem..

[5] Vanherck K., Koeckelberghs G., Vankelecom I.F.J. (2013). Crosslinking polyimides for membrane applications: A review. Prog. Polym. Sci..

[6] Sanders D.E., Smith Z.P., Guo R.L., Robeson L.M., McGrath J.E., Paul D.R., Freeman B.D. (2013). Energy-efficient polymeric gas separation membranes for a sustainable future: A review. Polymer.

[7] Higashihara T., Ueda M. (2015). Recent Progress in High Refractive Index Polymers. Macromolecules.

[8] Ding Y.C., Hou H.Q., Zhao Y., Zhu Z.T., Fong H. (2016). Electrospun polyimide nanofibers and their applications. Prog. Polym. Sci..

[9] Chu J.F., Liu Z.Y., Yang T., Kong A.G. (2023). Thioether-modified covalent aromatic polyimides with enhanced photocatalytic performance for hydrogen peroxide photosynthesis in pure water. Appl. Surf. Sci..

[10] Butnaru I., Constantin C.P., Damaceanu M.D. (2023). Optimization of triphenylamine-based polyimide structure towards molecular sensors for selective detection of heavy/transition metal ions. J. Photochem. Photobiol. A.

[11] Ounaies Z., Park C., Harrison J., Lillehei P. (2008). Evidence of piezoelectricity in SWNT-polyimide and SWNT-PZT-polyimide composites. J. Thermoplast. Compos. Mater..

[12] Jing H., Miao Z., Zeng Z., Liu H., Zhou S., Zou H., Liang M. (2022). Carbonization of graphene-doped isocyanate-based polyimide foams to achieve carbon foams with excellent electromagnetic interference shielding performance. Materials.

[13] Zheng L., Zhang K., Liu L., Xu F. (2022). Biomimetic architectured Kevlar/polyimide composites with ultra-light, superior anti-compressive and flame-retardant properties. Compos. Part B Eng..

[14] Zhang H., Fan X., Chen W., Wang Y., Liu C., Cui B., Li G., Song J., Zhao D., Wang D. (2022). A simple and green strategy for preparing flexible thermoplastic polyimide foams with exceptional mechanical, thermal-insulating properties, and temperature resistance for high-temperature lightweight composite sandwich structures. Compos. Part B Eng..

[15] Wang Y., Sun W., Yan D., Dai K., Li Z. (2021). Ultralight carbon nanotube/graphene/polyimide foam with heterogeneous interfaces for efficient electromagnetic interference shielding and electromagnetic wave absorption. Carbon.

[16] Yu L., Yu Y., Shi J., Zhang X., Gao F., Li C., Yang Z., Zhao J. (2022). Synthesis of a novel hyperbranched polyimide for reinforcing toughness and insulating properties of bismaleimide resin. Polymers.

[17] Zhang P., Liu H., Yao Y., Yang T., Sun J., Zhong X., Bao J., Zhao Y., Chen X. (2022). Preparation and properties of modified phenylethynyl terminated polyimide with neodymium oxide. Materials.

[18] Wang J., Yi X. (2003). Preparation and the properties of PMR-type polyimide composites with aluminum nitride. J. Appl. Polym. Sci..

[19] Chen F., Liu X., Liu H., Li S., Li S., Sun T., Zhao Y., Wang K. (2022). Improved interfacial performance of carbon fiber/polyetherimide composites by polyetherimide and modified graphene oxide complex emulsion type sizing agent. High Perform. Polym..

[20] Qiu Z., He F. (2001). Polyimide composites reinforced with whiskers. High Perform. Polym..

[21] Quirke N. (2001). Molecular modelling and simulation: Tools for the modern era. Mol. Simul..

[22] Barhaghi M.S., Crawford B., Schwing G., Hardy D.J., Stone J.E., Schwiebert L., Potoff J., Tajkhorshid E. (2022). py-MCMD: Python software for performing hybrid monte carlo/molecular dynamics simulations with GOMC and NAMD. J. Chem. Theory Comput..

[23] Batzner S., Musaelian A., Sun L., Geiger M., Mailoa J.P., Kornbluth M., Molinari N., Smidt T.E., Kozinsky B. (2022). E(3)-equivariant graph neural networks for data-efficient and accurate interatomic potentials. Nat. Commun..

[24] Chen C., Lu Y., Gao Y.M. (2021). Rapid boiling of argon vapor film confined by alternated hydrophobic and hydrophilic structures: Molecular dynamics study. Int. Commun. Heat Mass Transfer.

[25] Shen Z., Meng Z. (2022). Enhancing the efficiency of coal bed methane recovery by injecting carbon dioxide based on an anthracite coal macromolecular model and simulation methods. Energy Fuels.

[26] Li L., Li W., Gong J., Xu Y., Wu Z., Jiang Z., Cheng Y., Li Q., Ni H. (2021). An effective computational-screening strategy for simultaneously improving both catalytic activity and thermostability of alpha-l-rhamnosidase. Biotechnol. Bioeng..

[27] Chen K., Zeng K. (2022). Performance Optimization Model of Molecular Dynamics Simulation Based on Machine Learning and Data Mining Algorithm. Mobile Inf. Syst..

[28] Mo Y.Z., Zhang H., Xu J.C. Molecular dynamic simulation of the mechanical properties of polyimide based on Materials Studio. Proceedings of the International Conference on Mechanics and Materials Engineering (ICMME 2014).

[29] Qiu G., Ma W., Wu L. (2020). Thermoplastic and low dielectric constants polyimides based on BPADA-BAPP. Polym.-Plast. Technol. Mater..

[30] Lin J., Lin H., Yang W., Li X., Liu Y., Xie Z., Zhang P. Effect of different particle size of nano-zinc oxide on the surface binding energy in polyimide/zinc oxide composites. Proceedings of the 3rd International Conference on Chemical Engineering and Advanced Materials (CEAM 2013).

[31] Wen Q., Tang A., Chen C., Liu Y., Xiao C., Tan J., Li D. (2021). Synthesis, barrier performance, and molecular simulation of a high-barrier polyimide that contains amide groups. Mater. Res. Express.

[32] Fang X., Xie X.Q., Simone C.D., Stevens M.P., Scola D.A. (2000). A solid state 13C NMR study of the cure of 13C-labeled phenylethynyl end-capped polyimides. Macromolecues.

[33] Dufty J.W., Brey J.J. (2022). Green-Kubo expressions for a granular gas. J. Stat. Phys..

[34] Jaksic V., Ogata Y., Pillet C.A. (2006). The Green-Kubo formula and the Onsager reciprocity relations in quantum statistical mechanics. Commun. Math. Phys..

[35] Dal Cengio S., Levis D., Pagonabarraga I. (2019). Linear response theory and green-kubo relations for active matter. Phys. Rev. Lett..

[36] Ke Q., Gong X., Liao S., Duan C., Li L. (2022). Effects of thermostats/barostats on physical properties of liquids by molecular dynamics simulations. J. Mol. Liq..

[37] Rokni H., Lu W. (2020). Direct measurements of interfacial adhesion in 2D materials and van der Waals heterostructures in ambient air. Nat. Commun..

[38] Liu Y., Wu J., Wang Z., Lu X., Avdeev M., Shi S., Wang C., Yu T. (2020). Predicting creep rupture life of Ni-based single crystal superalloys using divide-and-conquer approach based machine learning. Acta Mater..

[39] Yoshikawa T., Doi T., Nakai H. (2019). Finite-temperature-based linear-scaling divide-and-conquer self-consistent field method for static electron correlation systems. Chem. Phys. Lett..

[40] Nevins D., Spera F.J. (2007). Accurate computation of shear viscosity from equilibrium molecular dynamics simulations. Mol. Simul..

