# Supplementary material for: The Relationship between Structure and Performance of Different Polyimides Based on Molecular Simulations
Source: Polymers (Basel). 2023 Jan 27;15(3):646. doi: 10.3390/polym15030646 (PMC9921807; doi:10.3390/polym15030646)
Supplement: Supplementary file 1 [file polymers-15-00646-s001.zip › polymers-2093096-supplementary.pdf]

The Relationship between Structure and Performance of Different Polyimides Based on Molecular Simulations

Supplementary Materials

Peng Zhang, Yadong Dai, Hansong Liu, Botao Dong, Yilun Yao, Jinsong Sun, Tao Yang, Xiangyu Zhong and Jianwen Bao

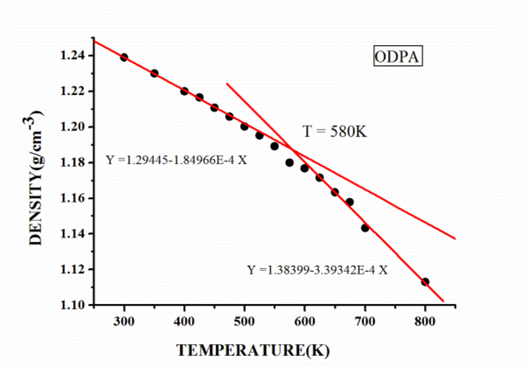

(**a**)

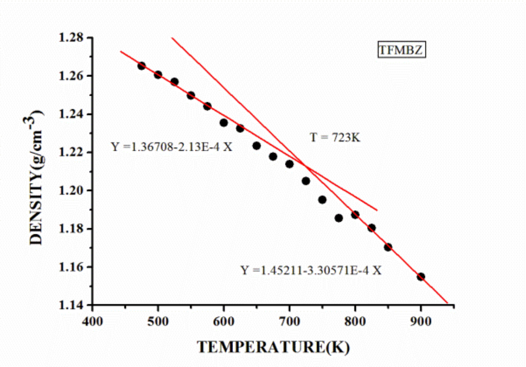

(**b**)

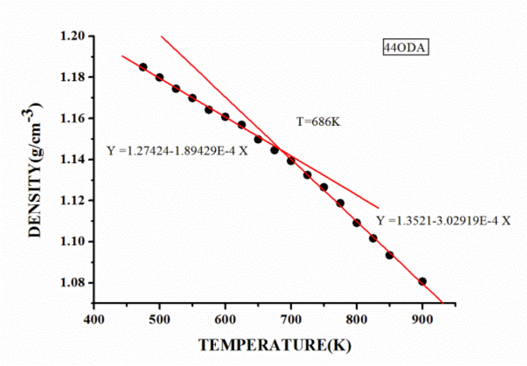

(**c**)

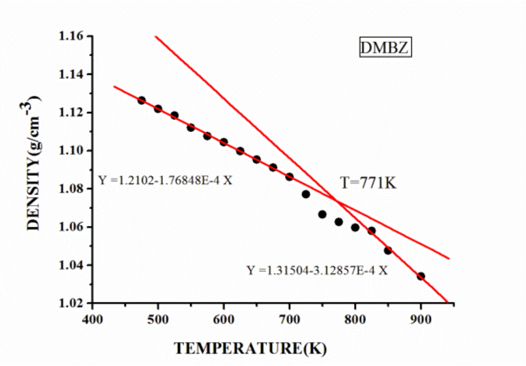

(d)

Figure S1. Linear fitting results of density variation with temperature of each simulated model. (**a**) ODPA-APB; (**b**) *α*-BPDA-TFMBZ; (**c**) *α*-BPDA- 4,4-ODA; (**d**) *α*-BPDA-DMBZ.

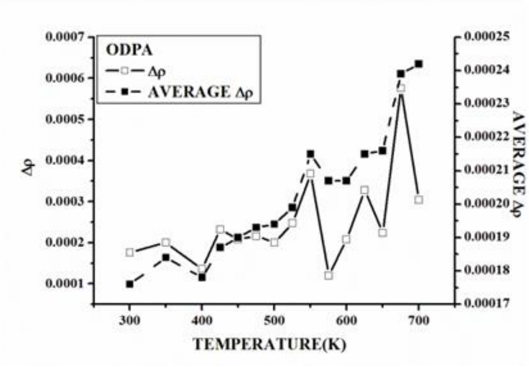

(**a**)

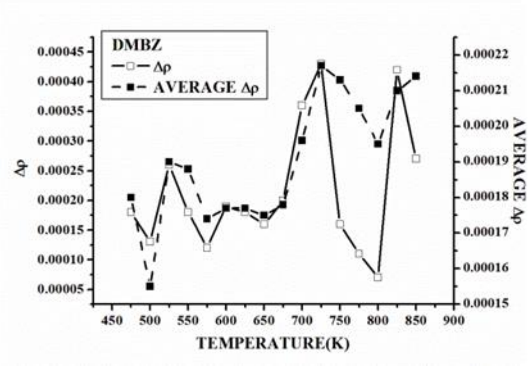

(**b**)

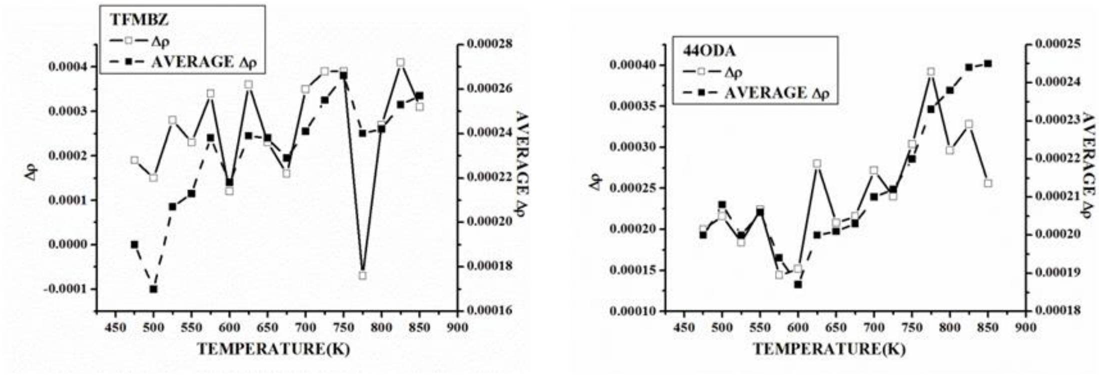

(**c**) (**d**)

Figure S2. *Δρ-T* curves and *Δρ* mean value curves with temperature of each system: (**a**) ODPA-APB; (**b**) α-BPDA-DMBZ; (**c**) α-BPDA-TFMBZ; (**d**) α-BPDA-4,4-ODA.

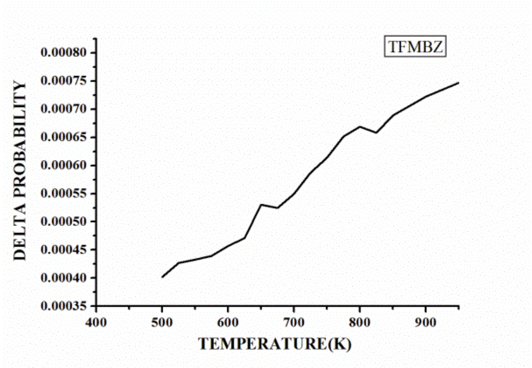

(**a**)

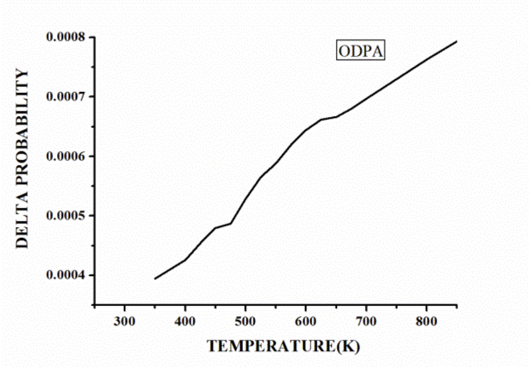

(**b**)

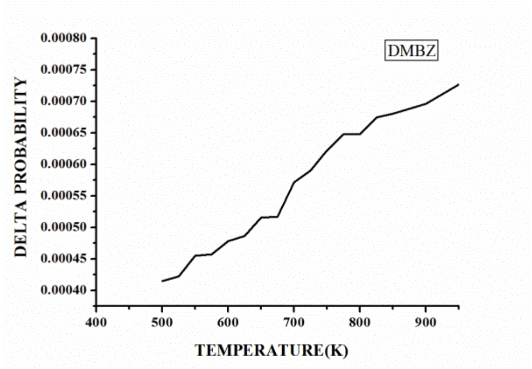

(**c**)

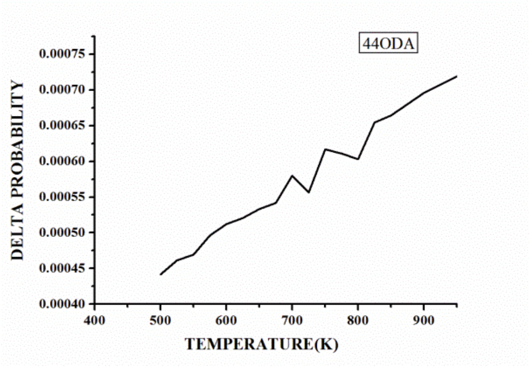

(**d**)

Figure S3. Temperature variation of dihedral angle distribution in main chain structure: (**a**) α-BPDA-TFMBZ; (**b**) ODPA-APB; (**c**) α-BPDA-DMBZ; (**d**) α-BPDA-4,4-ODA.
